# Prevalence of Sensorineural Hearing Loss in Children with Palliated or Repaired Congenital Heart Disease

**DOI:** 10.7759/cureus.6566

**Published:** 2020-01-04

**Authors:** Lalitha Gopineti, Mane Paulpillai, Andrea Rosenquist, Andrew H Van Bergen

**Affiliations:** 1 Pediatric Cardiology, Children's Mercy Hospitals and Clincs, Wichita, USA; 2 Pediatric Cardiology, Advocate Christ Medical Center, Oak Lawn, USA; 3 Pediatric Cardiology, Advocate Children’s Hospital, Oak Lawn, USA

**Keywords:** hearing loss, prevalence, congenital heart disease

## Abstract

Background

Children with congenital heart disease (CHD) are at increased risk of neurodevelopmental deficits, and the presence of sensorineural hearing loss (SNHL) may further lead to poor language skills acquisition and speech delays. Prevalence of SNHL in the general pediatric population is estimated to be 0.2% at birth to 0.35% during adolescence. Very few studies have attempted to estimate SNHL prevalence in children who have undergone congenital heart surgery.

Methods

This retrospective study aimed to estimate SNHL prevalence in children who underwent congenital heart surgery in our institution and were followed up in our high-risk pediatric cardiology clinics for four years from 2009 to 2013. Data were collected on demographics, preoperative variables, surgical variables, and post-operative variables.

Results

SNHL prevalence in asymptomatic, palliated/repaired CHD patients followed in our high-risk clinics and undergoing routine surveillance was 11.6% (20 of 172 patients with hearing impairment). SNHL prevalence was not statistically higher in single-ventricle patients (17.2%) compared to biventricular patients (14.7%). Inotropic score in the first 24 hours of postoperative period (p=0.05), lowest arterial PaO2 (p=0.003), duration of Lasix drip (p=0), and bolus dose in days (p=0.03) were all found to be statistically significant in the hearing-impaired group. However, using logistic regression, we identified no statistically significant predictors for hearing loss.

Conclusion

The results suggest the need for routine audiology screening of all patients with complex CHD, especially those who have undergone neonatal cardiac repair/palliation at less than one year of age, irrespective of risk factors.

## Introduction

Survival and life expectancy of children with congenital heart defects have dramatically increased in recent years due to advances in medical and surgical management strategies. Children with congenital heart disease (CHD) are at increased risk of neurodevelopmental deficits [1], and the presence of sensorineural hearing loss (SNHL) may further lead to poor language skills acquisition and speech delays [2]. The prevalence of SNHL in the general pediatric population is estimated to be 0.2% at birth to 0.35% during adolescence [3]. Many studies have estimated the prevalence of SNHL in the premature neonate population and have identified several associated risk factors [4]. One such study estimated the prevalence in neonates to be as high as 13.7% [5]. Very few studies, however, have attempted to estimate the prevalence of SNHL in children who have undergone congenital heart surgery. Of these studies, one old study estimated the prevalence to be 16% in CHD patients [6], and a recent study estimated the prevalence to be as high as 28.6% among childhood survivors after the Norwood/Sano operation only [7]. Currently, no clear recommendations exist for audiology screening in patients undergoing repair or palliation of CHD. The American Heart Association recommends audiology examination if there is no audiology examination since birth and if there is any suspicion of hearing impairment after cardiac surgery [8].

The first purpose of this study was to estimate the prevalence of SNHL in children who underwent congenital heart surgery in our institution and were followed up in our high-risk pediatric cardiology clinics for four years, from 2009 to 2013. The prevalence of SNHL in single-ventricle (SV) patients was also compared to that in biventricular (BiV) patients. Another objective of this study was to identify all possible risk factors associated with SNHL in palliated CHD patients to assess the need for routine hearing screening in patients with complex CHD.

## Materials and methods

We performed a retrospective chart review of patients followed in our high-risk pediatric cardiology clinics from 2009 to 2013 who had routine audiology evaluations. The study design was approved by the institutional review board. Data were collected on patient demographics and preoperative variables such as prematurity and birth weight, presence of genetic syndromes, as well as surgical variables such as total cardiopulmonary bypass time, circulatory arrest time, and cross-clamp time (Table 1).

**Table 1:** Demographic characteristics of patients CHD: congenital heart disease.

Characteristic	Hearing		Z statistic	P Value*
	Normal hearing (n=125)	Hearing loss (n=20)		
Sex				
Male	79 (63.2%)	15 (75%)	-1.023	0.307
Female	46 (36.8%)	5 (25%)		
Prematurity				
No	110 (88%)	15 (75%)	-1.560	0.119
Yes	15 (12%)	5 (25%)		
Diagnosis				
Mild CHD	7 (5.6%)	0	-1.089	0.276
Moderate 2V CHD	21 (16.8%)	0		
Severe 2V	33 (26.4%)	9 (45%)		
Palliated 1V	64 (51.2%)	11 (55%)		
Hearing loss status				
Moderate/Severe/Profound loss		10 (45%)	-11.967	0.000
Mild loss		11 (55%)		
Hearing aids status				
Yes		4 (20%)	-11.563	0.000
No		16 (80%)		
*Wilcoxon Mann-Whitney				

Post-operative variables were also collected. These included lowest arterial P^H^. and PaO2, exposure to known ototoxic medications such as furosemide, hydrochlorothiazide, gentamycin, and vancomycin and their cumulative doses in mg/kg along with duration, inotropic scores in the first two postoperative days, need for extracorporeal membrane oxygenation (ECMO), and clinical seizures. Data collection was performed by a research assistant who was blinded to the hearing test results. Table 2 shows various diagnoses in the study patients.

**Table 2:** Various diagnoses in the study patients CoA: coenzyme A; IAA: interrupted aortic arch; AS: aortic stenosis; DILV: double inlet left ventricle; DORV: double outlet right ventricle; DTGA: d-transposition of the great arteries; HLHS: hypoplastic left heart syndrome; PA: pulmonary atresia; IVS: intact ventricular septum; VSD: ventricular septal defect; L-TGA: levo-transposition of the great arteries; TAPVR: total anomalous pulmonary venous return; ASD: atrial septal defect; MV: mitral valve.

Diagnosis	Number of patients
Arch anomaly (CoA, IAA)	13
AS/sub AS	3
AVC/TOF	12
DILV	6
DORV	8
DTGA	15
Heterotaxy	7
HLHS	44
HRHS (PA/IVS, PA,VSD)	18
VSD	7
Other (L-TGA, TAPVR, Sinus venosus ASD, Coronary anomalies, Truncus, Isolated MV disease)	12

Exclusion criteria were any known genetic syndrome association with SNHL or family history of hearing loss, and any previously documented failed newborn screening. Drug dosages were calculated as cumulative doses in mg/kg based on the weight at the time of each operation. Audiology assessment was performed by an experienced pediatric audiologist using age-appropriate and standard procedures. Young children and developmentally delayed children underwent visually reinforced audiometry, while older children underwent behavioral audiological assessment using pure tone audiometry. All children with test results indicating impaired hearing underwent auditory brain stem response for confirmation. Hearing loss was classified as mild when the hearing loss range in decibels (dB) was 26-40 db. Hearing loss range in dB > 40 dB was grouped as moderate to severe to profound loss [9]. Comparisons between normal and abnormal hearing groups were done using the t test. All collected data were de-identified and stored in a password-protected computer database. All statistical tests were run using Statistical Package for the Social Sciences (SPSS Inc., Chicago, IL).

## Results

A total of 172 patients were identified, 20 of whom were found to have hearing impairment ranging from mild loss to moderate to severe to profound loss in one or both ears. No patient met the exclusion criteria. The prevalence of SNHL in asymptomatic; palliated/repaired CHD patients followed in our high-risk clinics undergoing routine surveillance was 11.6% (20 with hearing impairment out of 172 patients). The prevalence of SNHL was higher in SV patients (17.2%) compared to BiV patients (14.7%), but there was no statistically significant difference (odds ratio 1.16, confidence interval: 0.45-3) (Table 3). Twenty-seven out of 172 patients were eliminated from univariate analysis due to lack of records, as their surgeries were performed in a different institution. A total of 145 patients were reviewed for univariate analysis. Inotropic score in the first 24 hours of postoperative period (p= 0.05), lowest arterial PaO2 (p=0.003), the duration of furosemide drip (p=0), and bolus doses in days (p=0.03) were found to be statistically different between the two groups (Table 4). However, using logistic regression, we identified no statistically significant predictors for hearing loss.

**Table 3:** Prevalence of hearing loss in single-ventricle patients and biventricular patients SV: single-ventricle; BiV: biventricular; CI: confidence interval

	Hearing Loss	Normal Hearing	Prevalence
SV Pts	11	64	17.2%
BiV Pts	9	61	14.7%
Odds Ratio 1.16; CI 0.45-3			
Z statistic 0.32; p-value 0.75			

**Table 4:** Univariate analyses CPB: cardiopulmonary bypass; ECMO: extracorporeal membrane oxygenation.

	Normal Hearing (N=125)				Hearing Loss (N=20)					
	N	Mean	Median	Std. Deviation	N	Mean	Median	Std. Deviation	t Statistic	p value
Wks prematurity	13	35.115	35.700	1.361	5	35.140	36.000	2.332	-0.028	0.978
Birth Weight (Kg)	112	3.123	3.100	0.643	18	2.872	2.910	0.545	1.566	0.120
No. Cardiac Surgeries	125	2.272	2.000	1.058	20	2.450	2.000	0.887	-0.810	0.425
CPB time (min)	125	193.168	198.000	108.228	19	189.158	171.000	90.258	0.153	0.878
Arrest time (min)	125	24.816	0.000	32.226	19	23.368	0.000	26.243	0.186	0.852
Aortic CC time (min)	125	80.024	67.000	64.365	19	80.368	84.000	80.670	-0.021	0.983
VIS24	125	20.012	18.000	14.166	19	26.732	30.000	15.169	-1.909	0.058
VIS48	125	15.800	16.000	12.312	19	21.342	22.000	14.953	-1.775	0.078
Lowest Arterial Ph	120	7.241	7.250	0.097	19	7.196	7.220	0.095	1.867	0.064
Lowest Arterial PO2*	120	37.825	30.000	20.017	19	29.421	26.000	8.681	3.109	0.003
Number of Ventilator days	124	12.194	10.500	11.929	19	29.105	18.000	38.894	-1.882	0.076
Cumulative Lasix drip dose (mg/kg)	120	22.390	0.650	47.550	18	26.776	17.700	32.171	0.378	0.706
Total Lasix drip days*	121	54.160	4.000	107.811	18	7.167	4.500	7.950	4.710	0.000
Cumulative Lasix Bolus dose (mg/kg)	119	93.800	62.530	101.768	18	101.139	80.650	106.782	0.283	0.777
Total Lasix Bolus days*	124	11.742	8.500	11.665	18	24.967	19.500	23.719	-2.325	0.032
Cumulative Gentamycin dose (mgkg)	124	24.887	3.500	55.672	18	57.037	17.785	84.953	-1.558	0.136
Total Gentamycin days	124	6.250	0.000	24.168	18	13.167	4.000	18.875	-1.162	0.247
Cumulative Vancomycin dose (mgkg)	124	264.223	0.000	624.629	18	394.130	28.500	677.284	-0.816	0.416
Total Vancomycin days	124	3.597	0.000	6.351	19	11.842	2.000	19.909	-1.791	0.900
Cumulative Diuril dose (mgkg)	123	59.427	8.240	241.799	18	28.444	11.400	40.366	0.541	0.589
Total Diuril days	123	3.577	2.000	5.940	18	6.000	2.500	7.585	-1.557	0.122
Total ECMO days	125	0.280	0.000	1.3947	19	0.421	0.000	1.835	-0.393	0.695
* Significant difference between groups t-test										
Highlighted items with p-value less than 0.1 were included in the logistic regression.										

## Discussion

In our high-risk cohort, we estimated the prevalence of hearing loss of 11.6%, which is higher than in the general pediatric population as well as in neonatal intensive care unit (NICU) survivors (2%-4%) [10]. Limitations of the study include the small population size and the deficiency that not all patients underwent elective screening after congenital heart surgery. Only those patients followed up in high-risk pediatric cardiology clinics underwent the hearing test. Hence a true prevalence is not possible to calculate from the study. However, the study provides an estimate of prevalence which is higher than the prevalence of SNHL in the general pediatric population. This prevalence study did not examine all possible etiologies of SNHL like cytomegalovirus (CMV) infection, as no serologies were done to test for CMV infection due to this being a retrospective study. The biggest strength of this study is that the exposure to ototoxic medications was compared in the groups. The other advantage of our study is that it included all complexities of CHD, including mild CHD, moderate 2 ventricle, severe 2 ventricle, and palliated single ventricle, and could represent the estimated prevalence in repaired/palliated CHD in general.

Inner ear ischemia and hypo perfusion are the main proven physiological mechanisms for hearing loss after open heart surgery [11-12]. Hypoxia and bolus administration of furosemide have been reported to be significant risk factors for hearing loss in children after cardiopulmonary bypass surgery [7]. The duration of ECMO therapy and clinical seizure activity prior to ECMO are independently associated with SNHL [13].

## Conclusions

Our study supports the previously proven etiologies of hypoxia and longer duration of furosemide as strongly associated with SNHL. However, using logistic regression, we identified no single independent predictor as statistically significant for causing hearing loss in complex CHD. But because of the high prevalence of SNHL in this group, the results suggest the need for routine audiology screening of all patients with complex CHD, especially in patients who have undergone neonatal cardiac repair as well as palliation below one year of age, irrespective of risk factors. This screening is necessary to identify children at risk and intervene before SNHL negatively affects their neurodevelopment.
